# Handheld pH meter–assisted immunoassay for C-reactive protein using glucose oxidase–conjugated dendrimer loaded with platinum nanozymes

**DOI:** 10.1007/s00604-020-04687-9

**Published:** 2021-01-03

**Authors:** Bin Li, Lilin Ge, Peng Lyu, Meijuan Chen, Xiongfei Zhang, Shuping Xie, Qinan Wu, Hang Fai Kwok

**Affiliations:** 1 Collaborative Innovation Center of Chinese Medicinal Resources Industrialization, Nanjing University of Chinese Medicine, Nanjing, 210023 People’s Republic of China; 2 Institute of Translational Medicine, Faculty of Health Sciences, University of Macau, Avenida de Universidade, Taipa, Macau SAR; 3 College of Biological Science and Technology, Fuzhou University, Fuzhou, 350108 Fujian People’s Republic of China; 4 School of Medicine & Holistic Integrative Medicine, Nanjing University of Chinese Medicine, Nanjing, 210023 People’s Republic of China; 5 Division of Life Science, Hong Kong University of Science and Technology, Hong Kong, Hong Kong SAR

**Keywords:** Enzymatic cascade amplification, pH detection, Potentiometric immunoassay, Platinum nanozyme, Nanoparticle-encapsulated dendrimer, C-reactive protein

## Abstract

A simple and feasible pH meter–based immunoassay is reported for detection of C-reactive protein (CRP) using glucose oxidase (GOD)–conjugated dendrimer loaded with platinum nanozyme. Initially, platinum nanozymes were loaded into the dendrimers through an in situ synthetic method. Then, GOD and monoclonal anti-CRP antibody with a high molar ratio were covalently conjugated onto carboxylated dendrimers via typical carbodiimide coupling. The immunoreaction was carried out with a competitive mode in a CRP-coated microplate. Along with formation of immunocomplex, the added glucose was oxidized into gluconic acid and hydrogen peroxide by GOD, and the latter was further decomposed by platinum nanozyme, thus accelerating chemical reaction in the positive direction. The produced gluconic acid changed the pH of detection solution, which was determined using a handheld pH meter. Under optimum conditions, the pH meter–based immunoassay gave a good signal toward target CRP from 0.01 to 100 ng mL^−1^. The limit of detection was 5.9 pg mL^−1^. An intermediate precision ≤ 11.2% was acquired with batch-to-batch identification. No nonspecific adsorption was observed during a series of procedures to detect target CRP, and the cross-reaction against other biomarkers was very low. Importantly, our system gave well-matched results for analysis of human serum samples relative to a referenced ELISA kit.

Graphical abstract

**Supplementary Information:**

The online version contains supplementary material available at 10.1007/s00604-020-04687-9.

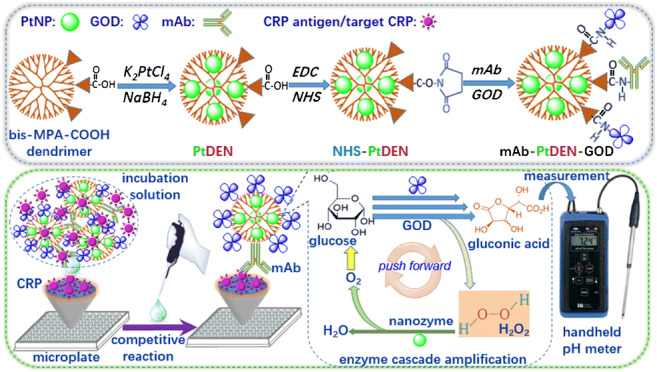

## Introduction

C-reactive protein (CRP), a spherical pentameric protein called acute phase reactants, goes up in response to inflammation. It displays several functions associated with host defense to promote agglutination and bacterial capsular swelling, and complement fixation through its calcium-dependent binding to phosphorylcholine [1]. CRP can interact with DNA and histones, and it may scavenge nuclear material released from damaged circulating cells. The concentration of CRP in plasma increases greatly during acute phase response to tissue injuries, infections, cancers, and renal and cardiovascular diseases [2]. It is induced by interleukin-1 and interleukin-6. These proteins are produced by white blood cells during inflammation [3]. Therefore, sensitive and specific detection of CRP would be advantageous for protein diagnostics.

Nowadays, analytical methods for CRP mainly involve immunoassays and aptasensing protocols. Immunoassays, based on specific antigen-antibody reaction, have gained increasing attention and become the dominant test tools in clinical diagnostics for disease-related proteins [4, 5]. Wu et al. developed a quantum dot–based immunoassay of CRP on a paper-based lateral flow test strip [6]. Broto et al. reported a nanoparticle-based bio-barcode assay for fluorescent detection of CRP in plasma samples [7]. Meyer et al. presented an immune-MALDI-MS approach for the quantification of CRP on reversed-phase tips [8]. Despite some advances in this field, there is still the requirement to simplify the assay procedures while preserving the essential benefits in sensitivity and specificity. In this regard, affordable medical diagnostics (i.e., point-of-care testing (POCT)) bring the patients for usage at bedside and the assays conveniently and immediately at the site of patient care [9, 10]. The pH meter is currently one of the most widely used electric devices to measure hydrogen-ion activity (acidity or alkalinity) in solution, thanks to its portable size, low cost, easy operation, and reliable quantitative results [11, 12]. Kwon et al. developed a pH meter–based immunoassay to detect the cardiac marker by using the labeled acetylcholinesterase with detection antibody for the hydrolysis of acetylcholine [11]. Chen et al. also constructed a pH meter–based potentiometric immunoassay by enzyme-conjugated hybridization chain reaction with two alternating hairpin DNA probes [13]. In these cases, the pH change of solution was derived from the labeled enzyme toward the catalysis/hydrolysis of substrates, and measured on a portable pH meter, thus reducing the difficulty of instrument operation [14, 15].

Another important issue for the development of the pH meter–based immunoassay with high efficiency is dependent on how to enhance sensitivity. Usually, diagnostic assay performance and practicality can be improved through engineering both the binding molecules and the reactions used to identify molecular binding events [16–19]. Dendrimers are well-defined and multivalent molecules with a highly branched three-dimensional nanometer-sized structure around an inner core with low polydispersity, and a high degree of functionality with abundant terminal groups [20]. Being a branched architecture, dendrimers have high surface to volume ratio [21]. Hydrophobic agents or inorganic nanoparticles can be easily loaded inside the cavity (void) in the dendrimer core [22, 23]. The presence of functional groups at the dendrimer surface provides the multivalent surface site for biomolecular conjugation via covalent/electrostatic interaction [24]. Typically, glucose oxidase (GOD) can oxidize glucose into gluconic acid and hydrogen peroxide [25]. Platinum nanoparticles (PtNPs) have been found to possess peroxidase-like activity [26, 27]. In the simultaneous presence of GOD and PtNPs, the added glucose molecules can convert into gluconic acid to maximum extent because PtNPs consume the produced hydrogen peroxide to push forward the oxidation reaction of glucose, thereby generating numerous gluconic acids. For this reason, our motivation in this study is to GOD-conjugate dendrimer loaded with PtNPs for the development of the pH meter–based immunoassay (Please see the detailed description on design of the pH meter–based immunosensing platform in the Supporting Information).

**Scheme 1:**
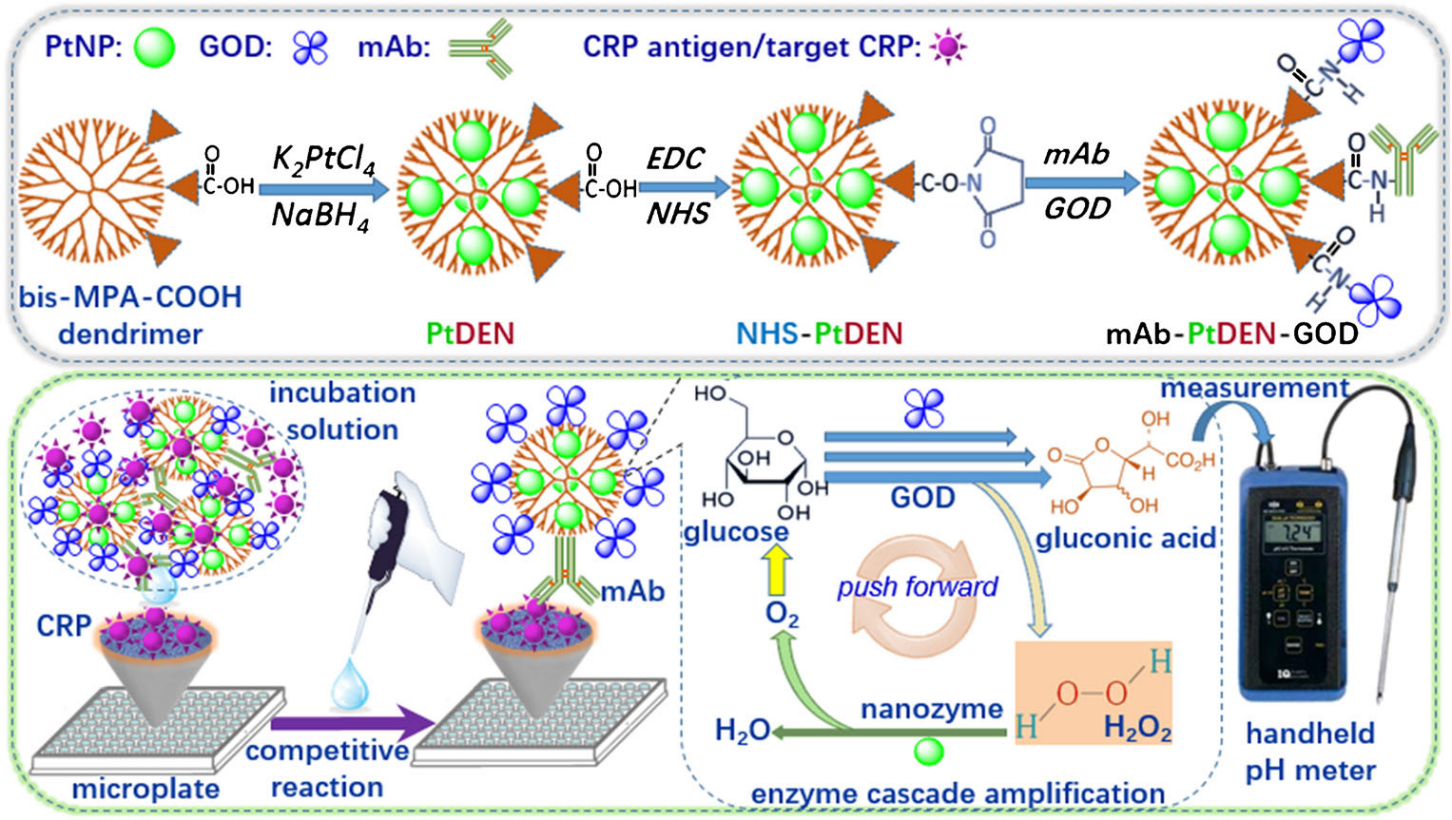
Schematic illustration of handheld pH meter–assisted immunoassay for detection of C-reactive protein (CRP) by using glucose oxidase (GOD) and monoclonal rabbit anti-human CRP antibody (mAb)–conjugated bis-MPA-COOH dendrimer encapsulated with platinum nanozyme (PtDEN): (top) fabrication process of mAb-PtDEN-GOD, and (bottom) competitive immunoreaction on CRP-coated microplate and enzymatic cascade reaction with pH meter readout

Herein, we design a simple and portable immunoassay for the sensitive detection of CRP with pH meter readout by GOD-conjugated dendrimer loaded with PtNPs (PtDEN-GOD) (Scheme 1). The immunoassay is implemented on CRP-coated microplates with a competitive reaction mode using anti-CRP antibody-labeled PtDEN-GOD as competitor. Subsequent pH detection of the as-produced gluconic acid in solution is conducted on a pH meter. The immunoassay combines high-loading dendrimer with GOD and platinum nanozyme for signal amplification. The objective of this work is to explore a new pH meter–assisted immunoassay for the cost-effective detection of low-abundance proteins with sensitivity enhancement.

## Materials and methods

### Bioconjugation of carboxylated PtDEN with GOD and antibody (mAb-PtDEN-GOD)

Before conjugation, PtNP-loaded bis-MPA-COOH dendrimer (denoted as PtDEN) was synthesized referring to previous report [28], and the detailed preparation process was described in the Supporting Information (note: bis-MPA-COOH = 2,2-bis(hydroxymethyl)propionic acid). The generation 4 (G4) bis-MPA-COOH dendrimer (trimethylol propane core) was used as an example for the preparation of PtDEN because its surface was functionalized with 48 carboxyl groups to facilitate the conjugation of the subsequent antibodies. Next, GOD and monoclonal rabbit anti-human C-reactive protein antibody (mAb) were conjugated to the PtDEN (denoted as mAb-PtDEN-GOD) through a typical carbodiimide coupling method [29]. Initially, NHS and EDC powders with an equal mass of 15 mg were simultaneously thrown into the above-prepared PtDEN suspension (2.0 mL), followed with continuous stirring (500 rpm) for 60 min at RT to activate the carboxyl groups on the surface of bis-MPA-COOH dendrimers. The resultant suspension was dialyzed using the abovementioned method (“Competitive immunoreaction and measurement on handheld pH meter”) to remove excess EDC and NHS for avoiding the subsequent cross-linkage between proteins. Thereafter, mAb antibody (0.5 mL, 200 μM) and GOD (0.5 mL, 600 μM) were added into the mixture and incubated for 6 h at 4 °C under slight stirring. Following that, the suspension was centrifuged for 10 min at 5000*g* to remove possibly produced precipitates during the reaction, and the collected supernatant was dialyzed as before. Finally, the obtained mAb-PtDEN-GOD was stored at 4 °C for further use. For comparison, other bioconjugates such as mAb-conjugated GOD (mAb-GOD), mAb-conjugated bis-MPA-COOH dendrimer (mAb-DEN), mAb and GOD-conjugated bis-MPA-COOH dendrimer (mAb-DEN-GOD), and GOD-conjugated PtDEN (PtDEN-GOD) were prepared by using the similar method. PtNP-labeled mAb antibody (mAb-PtNP) and PtNP-labeled mAb/GOD (mAb-PtNP-GOD) were prepared by direct reaction of antibody or GOD with PtNP.

### Competitive immunoreaction and measurement on handheld pH meter

Prior to measurement, CRP antigen-coated microplates were prepared as follows: (i) human C-reactive protein (50 μL per well, 10 μg mL^−1^) was added into a high-binding polystyrene 96-well microplate (cat# 655061, Greiner, Frickenhausen, Germany) and incubated overnight at 4 °C with adhesive plastics plate sealing film; and (ii) the microplate was incubated again with 300 μL per well of the blocking buffer (containing 1.0 wt% BSA in 10 mM pH 7.4 PBS) for 60 min at RT with slight shaking on a shaker after washing three times with washing buffer (containing 0.05% Tween 20, v/v, in 10 mM pH 7.4 PBS). The plate was then washed as before. Following that, 50 μL of CRP standard/sample and 50 μL of the above-prepared mAb-PtDEN-GOD were added to the well in turn and incubated for 60 min at RT with slight shaking to execute the competitive immunoreaction. The plate was washed again. One hundred microliters of PBS (10 mM, pH 6.5) containing 2.0 M glucose was injected to the well and reacted for 8 min at RT for enzyme cascade reaction. Finally, pH of the resultant solution was measured on a handheld pH meter. The obtained pH value was registered as signal of the immunoassay relative to different-level target CRP. All the measurements were carried out at room temperature (25 ± 1.0 °C). All the data referred to the average response of reaction with the corresponding standard deviation (mean ± SD) in triplicate, unless otherwise indicated. The sigmoidal curves were calculated by mathematically fitting experimental points using the Rodbard’s four-parameter function with Origin 6.0 software. Graphs were plotted in the form of pH against the logarithm of CRP concentration.

## Results and discussion

### Characterization of pH meter–based immunosensing platform

**Fig. 1:**
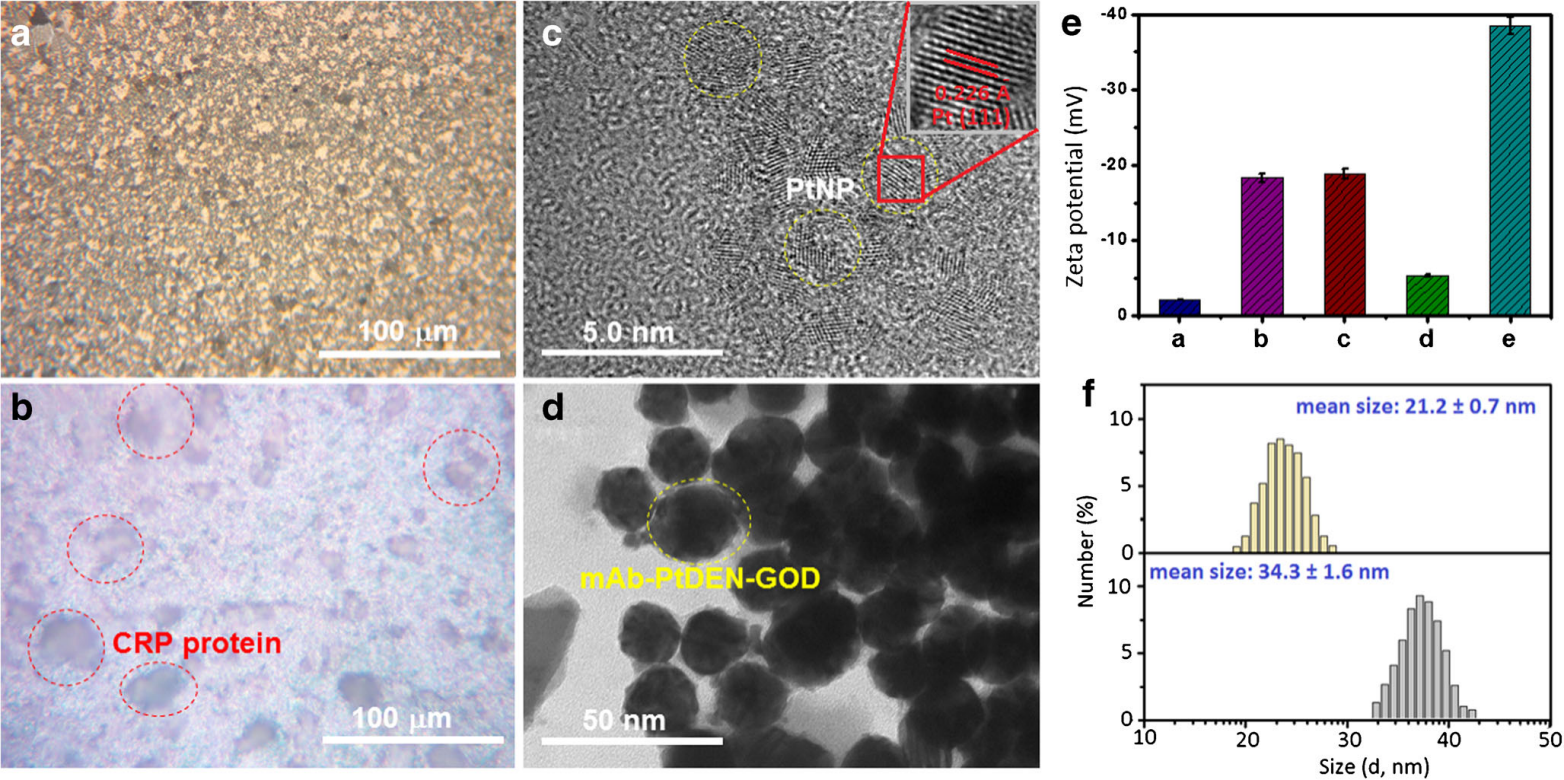
High-resolution inverted microscope images of **a** unmodified microplate and **b** CRP-coated microplate. **c** HRTEM image of PtDEN. **d** TEM image of mAb-PtDEN-GOD after negative staining. E zeta potentials of (a) PtNP, (b) bis-MPA-COOH dendrimer, (c) PtDEN, (d) EDC/NHS-activated PtDEN, and (e) mAb-PtDEN-GOD in ultrapure water. **f** DLS data of (top) PtDEN and (bottom) mAb-PtDEN-GOD

For the development of the pH meter–based immunoassay, the successful preparation of CRP-coated microplate and mAb-PtDEN-GOD should be characterized in detail. Since the surface topological structure of CRP-coated microplate was difficultly characterized directly through scanning electron microscopy and atomic force microscopy, we herein employed high-resolution inverted microscopy to observe the topological change of the microplate before and after modification with CRP proteins. Figure 1a gives typical micrograph of high-binding polystyrene microplate with a very rough surface, which was favorable for physical adsorption of proteins. As shown in Fig. 1b, a large number of proteins were attached on the surface of microplate after incubation with CRP antigens (note: BSA blocking was not done in this micrograph), indicating successful fabrication of CRP-coated plate.

As mentioned above, PtNPs were in situ synthesized in the bis-MPA-COOH dendrimer. Figure 1c shows high-resolution transmission electron microscope (HRTEM) of the as-synthesized PtDENs. It is found that many PtNPs were distributed in the dendrimers, and the average size of nanoparticles was ~ 2.3 nm in diameter. Moreover, we could also observe clearly the continuous lattice spacing of 0.226 nm corresponding to the (111) facet of the face-centered cubic (fcc) platinum crystal [30, 31]. Also, X-ray photoelectron spectroscopy (XPS) measurement was performed for PtDENs. As expected, the characteristic peaks at 284.1 eV, 530.1 eV, and 73.12 eV related to C 1s, O 1s, and Pt 4f core level regions of PtDENs, respectively, were observed in Fig. S1A-*a*, indicating the presence of Pt nanoparticles in the dendrimer. Unfavorably, HRTEM image of PtDEN did not give the nanostructures of dendrimers since it was a kind of organic molecules, which could not be observed at the high voltage. To tackle this shortcoming, the as-prepared mAb-PtDEN-GOD conjugates were characterized by TEM after negative staining with sodium phosphotungstate (2.0 wt%, pH 7.3) (note: not good for negative staining of dendrimers). As seen from Fig. 1d, a layer of translucent structures was coated on the nearly spherical dendrimers, and the mean size was ~ 28 nm in diameter. Logically, one puzzling question arises as to whether mAb antibodies and GOD molecules were really conjugated onto the dendrimers through the carbodiimide coupling. To demonstrate this issue, we used dynamic light scattering (DLS) to monitor zeta potentials and sizes of PtDEN after reaction with EDC/NHS and mAb antibody, respectively (note: No obvious difference from characteristic peaks between PtDEN and mAb-PtDEN-GOD with Fourier transform infrared spectroscopy (FTIR) because they contained C=O, N–H, and C–N bond, Fig. S1B). Pure PtNP (*ξ* = − 2.1 mV, Fig. 1ea) and bis-MPA-COOH dendrimer (*ξ* = − 18.3 mV, Fig. 1eb) exhibited a negatively charged species. In contrast, the zeta potential of the synthesized PtDEN (*ξ* = − 18.8 mV, Fig. 1ec) was slightly lower than that of bis-MPA-COOH dendrimer (Fig. 1eb). When coupling carbodiimide of PtDEN with EDC/NHS, however, the potential increased to − 5.4 mV (Fig. 1ed), thanks to introduction of non-charged NHS molecules. Significantly, the zeta potential heavily decreased to − 38.5 mV (Fig. 1ee) after EDC-functionalized PtDEN further reacted with GOD and mAb antibody. The reason was ascribed to the fact that the isoelectric points of GOD and anti-CRP antibody were ~ pH 4.6 and ~ pH 5.7 (which were measured by capillary isoelectric focusing electrophoresis, respectively), and they had negative charges in pH 7.0 ultrapure water. Moreover, the size of mAb-PtDEN-GOD (34.3 ± 1.6 nm) (Fig. 1f, bottom) was obviously more than that of PtDENs (21.2 ± 0.7 nm) (Fig. 1f, top) on the basis of DLS data (note: The sizes of PtDEN were almost the same before and after reaction with EDC/NHS, data not shown), and the increasing size mainly derived from the labeled biomolecules. Furthermore, another two characteristic peaks for N1s and S2p for the proteins could be observed from the XPS data (Fig. S1A-*b*). These results preliminarily revealed the formation of mAb-PtDEN-GOD.

### Characteristics of the signal amplification and control tests

**Fig. 2:**
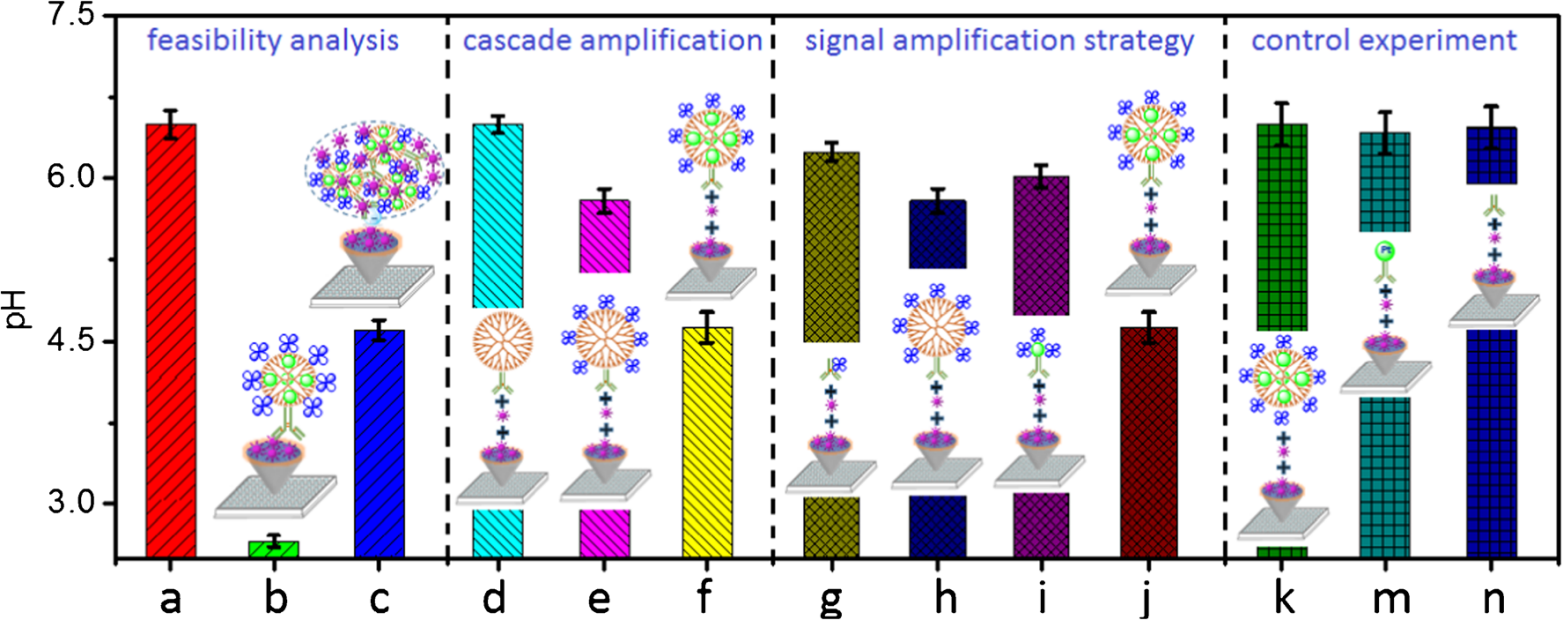
Signal readouts of **a** pH 6.5 PBS (10 mM), and (b, c) pH meter–based immunoassay in the absence (b) and presence **c** of 1.0 ng mL^−1^ CRP with mAb-PtDEN-GOD. (d–n) Signal readouts of pH meter–based immunoassay in the presence of 1.0 ng mL^−1^ CRP using differently labeled probes: **d** mAb-DEN; **e** mAb-DEN-GOD; (**f**, **j**) mAb-PtDEN-GOD; **g** mAb-GOD; **h** mAb-DEN-GOD; **i** mAb-PtNP-GOD; **k** PtDEN-GOD; **m** mAb-PtNP; and **n** mAb. All immunoreactions were carried out on CRP-coated microplates, and the final pH measurement was executed in PBS (100 μL, 10 mM, pH 6.5) containing 2.0 M glucose. Each data point represents the average value obtained from three measurements, and the error bars stand for 95% confidence interval of the mean for pH value

By using the as-prepared mAb-PtDEN-GOD and CRP-coated microplate, we first evaluated the feasibility of the pH meter–based immunoassay for the detection of target CRP (1.0 ng mL^−1^ used as an example) with a competitive-type format. Figure 2a represents the background signal of pH 6.5 PBS. Obviously, the presence of target CRP could cause the pH change of the detection solution (Fig. 2c) in comparison with zero analyte (Fig. 2b). The pH shift in the presence of target CRP was ascribed to the competitive immunoreaction between target CRP and the coated CRP on the microplate for the labeled mAb on mAb-PtDEN-GOD, thus decreasing the captured amount of mAb-PtDEN-GOD on the microplate. These results indicated that the pH meter–based immunoassay was feasible for the detection of target CRP on CRP-coated microplate by using mAb-PtDEN-GOD as the signal tag.

As described above, the as-synthesized mAb-PtDEN-GOD had the cascade signal amplification. To clarify this issue, a comparative study was carried out by using differently labeled probes (i.e., mAb-DEN, mAb-DEN-GOD, and mAb-PtDEN-GOD) for the detection of 1.0 ng mL^−1^ CRP on CRP-coated microplate. The immunoassay of using mAb-DEN (Fig. 2d) gave almost the same pH signal as that of pH 6.5 PBS (Fig. 2a), indicating that the formed immunocomplex with mAb-DEN could not change the pH of detection solution. Significantly, pH of detection solution gradually decreased when using mAb-DEN-GOD (Fig. 2e) and mAb-PtDEN-GOD (Fig. 2f) as the signal tags. The reason was ascribed to the fact that the added glucose molecules were first oxidized to gluconic acid and hydrogen peroxide (H_2_O_2_) by the conjugated GOD in the presence of oxygen, and then, the produced H_2_O_2_ was reduced to hydrogen oxide (H_2_O) and oxygen (O_2_) via platinum nanozyme with peroxidase-like activity, thus pushing glucose oxidation forward to generate numerous gluconic acid molecules with the signal amplification (Please see the detailed discussion on the role of platinum nanoparticles, glucose oxidation reaction process with GOD and PtNP, and comparative studies of differently labeled probes for the signal amplification in the Supporting Information, Fig. S2-S5).

Except for the cascade signal amplification by GOD and the encapsulated PtNP in the dendrimer, we also monitored the advantages of bis-MPA-COOH dendrimers by comparing with four labeled probes: mAb-GOD, mAb-DEN-GOD, mAb-PtNP-GOD, and mAb-PtDEN-GOD. It is found that pH variations (relative to pH 6.5 PBS, Fig. 2a) of using mAb-DEN-GOD (Fig. 2h) and mAb-PtDEN-GOD (Fig. 2j) were more than those of mAb-GOD (Fig. 2(g)) and mAb-PtNP-GOD (Fig. 2i), suggesting that introduction of the dendrimers could amplify the detectable signal (Please see the detailed discussion on the role of dendrimer in the Supporting Information, Fig. S6). As the control tests, PtDEN-GOD (Fig. 2k), mAb-PtNP (Fig. 2m), and mAb (Fig. 2n) were employed as the signal tags for detection of 1.0 ng mL^−1^ CRP on CRP-coated microplate, respectively. Almost no pH variations were observed in these cases relative to pH 6.5 background signal (Fig. 2a). Furthermore, we also found that the cascade reaction could not be fulfilled in the absence of GOD, even if platinum nanozyme was labeled to mAb antibody (Fig. 2m). On the basis of these results, we could clearly confirm that the synthesized mAb-PtDEN-GOD could be utilized as the signal tag to amplify the signal of the pH meter–based immunoassay.

### Calibration plots of pH meter–based immunoassay toward target CRP standards

**Fig. 3:**
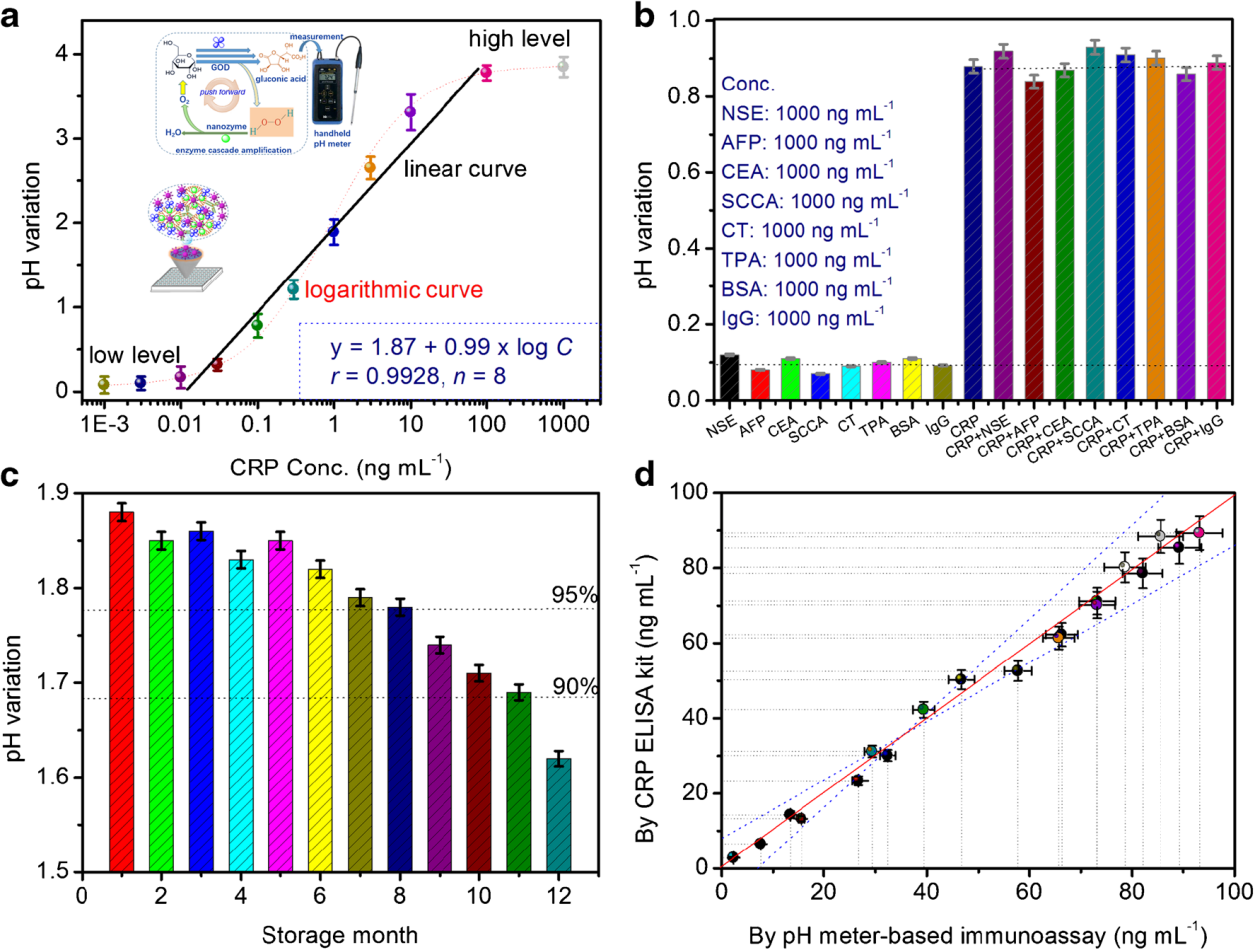
**a** Calibration plots of pH meter–based immunoassay toward different-concentration CRP standards with a competitive-type assay format by using mAb-PtDEN-GOD as the competitor in PBS (100 μL, 10 mM, pH 6.5) containing 2.0 M glucose. **b** The specificity of pH meter–based immunoassay against 0.1 ng mL^−1^ CRP, 1000 ng mL^−1^ NSE, 1000 ng mL^−1^ AFP, 1000 ng mL^−1^ CEA, 1000 ng mL^−1^ SCCA, 1000 ng mL^−1^ CT, 1000 ng mL^−1^ TPA, 1000 ng mL^−1^ BSA, and 1000 ng mL^−1^ IgG. **c** The storage stability of CRP-coated microplate and mAb-PtDEN-GOD. **d** Method accuracy for analysis of human serum specimens between pH meter–based immunoassay and commercial CRP ELISA kit. Each data point represents the average value obtained from three measurements (*n* = 3), and the error bars stand for the 95% confidence interval of the mean for pH variation

Under the optimum conditions (Please see the relative description in the Supporting Information, Fig. S7), CRP-coated microplate and mAb-PtDEN-GOD were utilized to determine CRP standards with different concentrations on a handheld pH meter with a competitive immunoassay mode. Figure 3a shows pH variations of the pH meter–based immunoassay relative to the decimal logarithm of CRP concentrations within the dynamic range of 0.001–1000 ng mL^−1^. Obviously, pH variations were very small at high and low levels of 100–1000 ng mL^−1^ and 0.001–0.01 ng mL^−1^. A good linear relationship was acquired in the concentration range from 0.01 to 100 ng mL^−1^ with a detection limit (LOD) of 5.9 pg mL^−1^ and a limit of quantification (LOQ) of 19.7 pg mL^−1^ at signal-to-noise ratios of 3*σ* and 10*σ*, respectively (where *σ* is the standard deviation of a blank solution, *n* = 11). The regression equation could be fit as *y* (ΔpH) = 1.87 + 0.99 × log*C*_[CRP]_ (ng mL^−1^, *r* = 0.9928, *n* = 8). The sensitivity of the pH meter–based immunoassay was 9.77 pH ng/mL. Moreover, the linear range and limit of detection of the pH meter–based immunoassay were comparable with those of other CRP assay methods (Table 1). Though the LOD of our strategy was higher than those of partial electrochemical systems, the developed pH meter–based immunoassay did not need expensive instrumentations, and professional/technical personnel. Significantly, our system was capable of continuously performed all steps within ≤ 70 min for one sample, including incubation, washing and pH measurement, which was less than for commercial CRP ELISA kit (approximately 3.5 h). Meanwhile, the pH meter–based immunoassay was relatively simple and low-cost without the expensive instruments and complex operation (~USD $1.93 for a single test versus ~ USD $7.81 per sample with commercialized available human CRP ELISA kit from Sigma-Aldrich Product cat# no.: RAB0096).

**Table 1:** Comparison of pH meter–based immunoassay with other CRP detection methods on analytical properties

Method	Linear range	LOD	Ref.
Colorimetric aptamer assay	0.889–20.7 μg mL^−1^	1.2 μg mL^−1^	[32]
Biophotonic assay	2.0–14.7 μg mL^−1^	0.42 μg mL^−1^	[33]
Electrochemical immunosensor	0.01–100 ng mL^−1^	3.3 pg mL^−1^	[34]
Capacitive immunoassay	0.25–2.0 μg mL^−1^	0.5 μg mL^−1^	[35]
Impedimetric immunoassay	0.2–5.0 μg mL^−1^	3.7 pg mL^−1^	[36]
Electrochemiluminescence immunosensor	0.05–6.25 ng mL^−1^	11 pg mL^−1^	[37]
Impedimetric immunosensor	0.1–10 ng mL^−1^	0.1 ng mL^−1^	[38]
Fluorescent immunodipsticks	0.5–1000 ng mL^−1^	0.3 ng mL^−1^	[6]
Electrochemical immunoassay	0.05–100 μg mL^−1^	15 ng mL^−1^	[39]
pH meter–based immunoassay	0.01–100 ng mL^−1^	5.9 pg mL^−1^	This work

### Reproducibility, specificity, and storage stability

To investigate the reproducibility of the pH meter–based immunoassays, CRP-coated microplate and mAb-PtDEN-GOD with the same batch or different batches were used for the determination of three CRP levels (i.e., 0.01, 1.0, and 100 ng mL^−1^ used in this case) because the immunoassay could not be repeatedly used (disposable). The coefficients of variation (CVs) for the intra-assays were 5.8%, 3.4%, and 7.1% (*n* = 3) for 0.01, 1.0, and 100 ng mL^−1^, respectively, whereas those for the inter-assays were 10.4%, 9.8%, and 11.2% (*n* = 3) for the abovementioned levels. Hence, the reproducibility and precision of the pH meter–based immunoassays were acceptable.

The specificity of the pH meter–based immunoassay was studied by analyzing other proteins or biomarkers possibly present in human serum, e.g., neuron-specific enolase (NSE), alpha-fetoprotein (AFP), carcinoembryonic antigen (CEA), squamous cell carcinoma antigen (SCCA), calcitonin (CT), tissue polypeptide antigen (TPA), BSA, and IgG. The evaluation was carried out by assaying the non-targets alone or mixture containing CRP and non-target. The comparison was performed by observing the effect of high-concentration non-targets on the low-level CRP. As shown in Fig. 3b, pH variations of non-targets were close to zero, and strong pH variations could be observed in the presence of CRP. Compared with pure CRP alone, introduction of non-targets with target CRP did not cause the significant pH variations, thus suggesting high selectivity and specificity.

The storage stability of CRP-coated microplates and mAb-PtDEN-GOD were monitored over a 1-year period at 4 °C. After every 1 month, they were taken out to measure target CRP (1.0 ng mL^−1^ used as an example). As seen from Fig. 3c, pH variations could maintain ≥ 95% of the initial signal within 8 months. After storing for 11 months, the signal could also preserve more than 90%. Such long-term storage stability mainly stemmed from covalent conjugation of mAb and GOD with PtDEN and high-binding polystyrene microplate.

### Analysis of human serum specimens

To investigate the accuracy of the pH meter–based immunoassay, we collected 20 human serum specimens containing target CRP from the hospital of our University. All the experiments were performed in compliance with the relevant laws and Guidelines of Nanjing University of Chinese Medicine (China), and the experiments have been approved. Informed consent was obtained for any experimentation with human subjects. Prior to measurement, these serum samples were initially centrifuged for 10 min at 5000*g* to remove the possible precipitates, and then were determined by using the pH meter–based immunoassay. As the reference, the obtained results were compared with those of using commercial CRP ELISA kits (Fig. 3d). As shown in Fig. 3d, the data from two methods were fit to a regression equation as follows: *y* = 0.0698 + 0.9733*x* (*r* = 0.9917, *n* = 20). The slope and intercept of the regression equation were close to ideal “0” and “1,” respectively [40, 41]. Therefore, almost no significant differences at the 0.05 significance level were encountered for the analysis of 20 human serum specimens between two methods, indicating good accuracy.

## Conclusions

This study successfully developed a simple and easy-operation immunoassay for the quantitative screening of C-reactive protein on a handheld pH meter. Experimental results indicated that the pH meter–based immunoassay had high sensitivity, good reproducibility, high specificity, and long-term storage stability. Combination of natural enzyme with nanozyme can efficiently promote the adequate catalysis of the substrates, thus facilitating the formation of the products. Moreover, one-step immunoreaction with one-kind antibody reduces the assay time and decrease the assay cost. Furthermore, the pH meter–based immunoassay with the coated microplates can be used in the miniaturized devices by using the portable pH meters, thus opening new opportunities for the protein diagnostics and biosecurity. Nevertheless, one disadvantage of our strategy is that the incubation time for target CRP is relatively long during the competitive immunoreaction. Therefore, future work should focus on improvement of reaction systems or reactive conditions.

## Supplementary information

ESM 1 (DOCX 1536 kb)
