# Supplementary material for: Handheld pH meter–assisted immunoassay for C-reactive protein using glucose oxidase–conjugated dendrimer loaded with platinum nanozymes
Source: Mikrochim Acta. 2021 Jan 3;188(1):14. doi: 10.1007/s00604-020-04687-9 (PMC7779416; doi:10.1007/s00604-020-04687-9)
Supplement: Supplementary file 1 — (DOCX 1536 kb) [file 604_2020_4687_MOESM1_ESM.docx]

**ELECTRONIC SUPPORTING MATERIAL**

**Handheld pH meter-assisted immunoassay for C-reactive protein using glucose oxidase-conjugated dendrimer loaded with platinum nanozymes**

Bin Li,^1,2,†^ Lilin Ge,^1^**,***^,†^ Peng Lyu,^3^ Meijuan Chen,^4^ Xiongfei Zhang,^4^ Shuping Xie,^1,5^ Qinan Wu,^1^ and Hang Fai Kwok^2,^*

^1^ Collaborative Innovation Center of Chinese Medicinal Resources Industrialization, Nanjing University of Chinese Medicine, Nanjing, 210023, People’s Republic of China

^2^ Institute of Translational Medicine, Faculty of Health Sciences, University of Macau, Avenida de Universidade, Taipa, Macau SAR

^3^ College of Biological Science and Technology, Fuzhou University, Fuzhou, Fujian 350108, People’s Republic of China
^4^ School of Medicine & Holistic Integrative Medicine, Nanjing University of Chinese Medicine, Nanjing 210023, People’s Republic of China
^5^ Division of Life Science, Hong Kong University of Science and Technology, Hong Kong SAR

**CORRESPONDING AUTHOR INFORMATION**

*E-mail addresses:* gelilin@njucm.edu.cn (L.L. Ge) & hfkwok@um.edu.mo (H.F. Kwok).

† Bin Li and Lilin Ge have contributed equally to this work.

**TABLE OF CONTENTS**

S1. Materials and methods S3

S1.1. Chemical and reagentS3

S1.2. Preparation of platinum nanoparticles-loaded bis-MPA-COOH dendrimer (PtDEN) S3

S1.3. Human CRP ELISA assay for real samples S4

S2. Partial results and discussionS5

S.2.1. Design of pH meter-based immunosensing platformS5

Fig. S1S6

S2.2. Elucidation of the role of platinum nanoparticlesS6

Fig. S2S6

Fig. S3S8

Fig. S4S9

S2.3. Comparative studies of differently labelling strategies with mAb antibodyS10

*Role of PtNP and GOD*S10

Fig. S5S11

*Role of dendrimer*S11

Fig. S6S13

S2.4. Investigation of substrate effectsS13

Fig. S7S14

S2.5. Optimization of experimental conditionsS15

Fig. S8S16

ReferenceS18

**S1. MATERIALS AND METHODS**

**S1.1. Material and Reagent**

Human C-reactive protein (CRP; recombinant, expressed in *E. coli*, liquid in 20 mM Tris-HCl buffer containing 2.0 mM CaCl_2_/0.14 M NaCl/0.05% NaN_3_, pH 7.5; cat# no.: C1617, 1.0 mg mL^-1^), bis-MPA-COOH dendrimer (trimethylol propane core, generation 4, functionality: carboxyl surface group, surface group no. 48), glucose oxidase (GOD) from *Aspergillus niger* (type VII, lyophilized powder, ≥100,000 units/g solid without added oxygen), bovine serum albumin (BSA; Vetec^TM^ reagent grade, lyophilized powder, ≥98%), K_2_PtCl_4_ (≥99.9% trace metals basis, powder, Pt ≥46.2%) and NaBH_4_ (granular, 99.99% trace metals basis) were purchased from Sigma-Aldrich (Shanghai, China). Monoclonal rabbit anti-human C-reactive protein antibody (clone Y284; reactivity: human; cat# no.: ab32412) and human CRP ELSIA kits (cat# no.: ab181416; CV: 7.2%, *n* = 3; sensitivity: 4.0 pg mL^-1^; linear range: 15.63 – 1000 pg mL^-1^; recovery: 90.1%; one-step assay within 90 min) were obtained from Abcam (Hongkong, China) (note: This antibody reacted with an epitope located in the C terminal region of C-reactive protein). 1-Ethyl-3-(3-dimethyl-aminopropyl) carbodiimide hydrochloride (EDC) and *N*-hydroxysulfosuccinimide (NHS) were gotten from Aladdin (Shanghai, China). All the other reagents were of analytical grade. Double distilled water was used in all runs. All buffers used in this study, including phosphate-buffered saline (PBS) solution and Tris-HCl buffer, were the products of Sigma-Aldrich.

**S1.2. Preparation of Platinum Nanoparticles-Loaded bis-MPA-COOH Dendrimer (PtDEN)**

Initially, K_2_PtCl_4_ aqueous solution (2.0 mL, 5.0 mM) was quickly added to bis-MPA-COOH dendrimer aqueous solution (0.01 mM, 10 mL), and the pH of the mixture was then adjusted to ~5.0 with HCl. After stirring for 3 days (150 rpm) in the dark at room temperature (RT), 11.5 mg of NaBH_4_ was thrown in the mixture under the protection of nitrogen, and further reacted overnight under the same conditions. During the process, Pt(II) ions were *in-situ* reduced to zero platinum nanoparticles. To remove the impurities including free PtNPs and excess chemicals, the resulting suspension was dialyzed in a dialysis bag (BioSharp, mw cut-off 3,500 Da) against ultrapure water (pH 5.0 – 6.0, adjusted with HCl) at RT in a dark for 24 h by changing ultrapure water every 6 h. Finally, carboxylated PtDENs were used for conjugation of biomolecules, or stored at RT in a dark when not in use. As control tests, pure PtNPs (~20 nm in diameter) without the dendrimer were also prepared similarly by using K_2_PtCl_4_ and NaBH_4_.

**S1.3. Human CRP ELISA Assay for Real Samples**

For reference, plasma CRP concentrations were determined by using human CRP ELISA kits. In a typical target CRP detection experiment, samples or standards were added to the wells, followed by antibody mix. After incubation, the wells were washed to remove unbound material. TMB substrate was added and during incubation was catalyzed by horseradish peroxidase, generating blue coloration. The enzymatic reaction was stopped by adding 50 μL of 2.0 M H_2_SO_4_ to each well. The results of ELISA were measured by a spectrophotometric ELISA reader at a wavelength of 450 nm.

**S2. PARTIAL RESULTS AND DISSCUSSION**

**S2.1. Design of pH Meter-Based Immunosensing Platform**

As a newly developed detection protocol, a simple, low-cost, sensitive and specific immunoassay would be very overwhelming in modern healthcare to prevent wide spread of diseases and facilitate personalized medicine. In this work, the immunoreaction is carried out with a one-step competitive format in the low-cost microplates, whereas the measurable signal is quantitatively achieved on a handhold pH meter. C-reactive proteins are immobilized on the wells through physical adsorption. Platinum nanoparticles are encapsulated in the dendrimers on the basis of *in-situ* reduction reaction. GOD and rabbit anti-human CRP monoclonal antibody are covalently conjugated onto carboxylated dendrimer *via* carbodiimide coupling. In the present of target CRP, the analyte competes with the immobilized CRP in the plate for the labeled antibody on the dendrimer, and target analyte can be detected from the signal resulted from the labels. Relative to the sandwiched assay, the competitive immunoassay is simpler and reduces the incubation time. Moreover, monoclonal anti-CRP antibody offers several advantages including high batch-to-batch consistency and reproducibility, improved sensitivity and specificity, long-term security of supply, and animal-free production according to the product datasheet from Abcam. In addition, the signal amplification is very crucial for obtaining a low limit of detection (LOD) and quantification (LOQ). To realized our design, there are two basic concerns for this purpose in this study: (i) introduction of multiple surface groups on the dendrimers is expected to increase the conjugation amount of natural enzymes; and (ii) utilization of bienzyme including GOD and platinum nanozyme can push enzymatic cascade reaction forward to maximize its own production (gluconic acid), thus resulting in the signal amplification with high sensitivity.

*
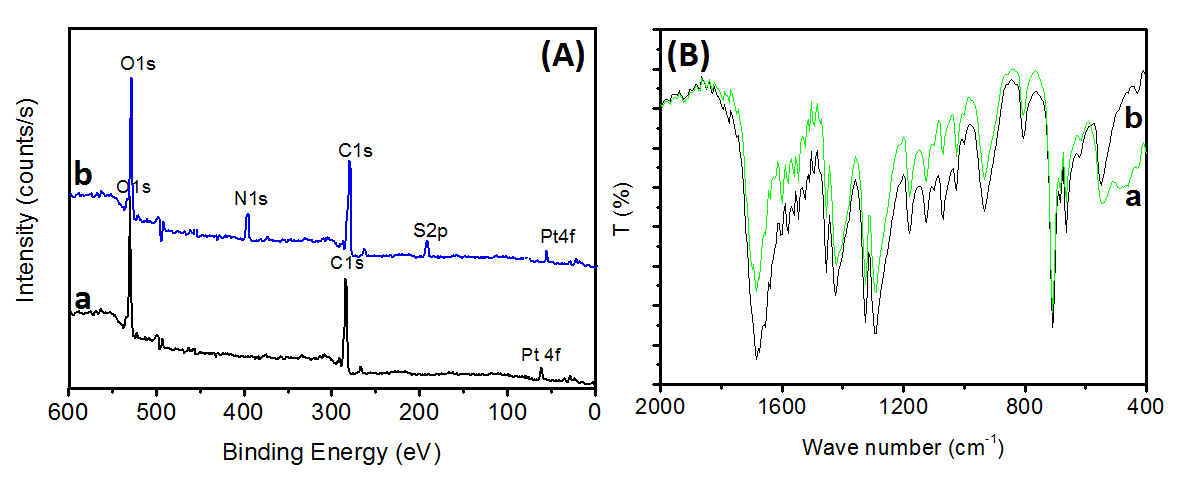
*

**Fig. S1 A** XPS analyses and **B** FTIR spectra of the as-synthesized ***a*** PtDENs and ***b*** mAb-PtDEN-GOD.

**S2.2. Elucidation of the Role of Platinum Nanoparticles (PtNPs)**

In this work, platinum nanoparticles mainly act as peroxidase-like mimics to reduce the peroxide peroxide into hydrogen oxide and oxygen. To clarify this issue, we first used cyclic voltammetry to investigate the peroxidase-like activity of platinum nanoparticles in PBS (pH 6.5, 10 mM). Prior to experiment, platinum nanoparticles (~20 nm in diameter) were modified on a cleaned glassy carbon electrode (GCE) by mixing PtNPs with (0.3 *wt* %) chitosan colloids in the acetic acid according to the literature [1]. Thereafter, the formed PtNP-chitosan colloids were dropped onto the GCE, and dried at room temperature. Cyclic voltammograms were measured in PBS (pH 6.5, 10 mM) at 50 mV s^-1^ (*vs*. Ag/AgCl) from -0.4 V to +0.6 mV (Fig. S2-A). Curve '*a*' shows cyclic voltammogram of PtNP-chitosan-modified GCE in PBS (pH 6.5, 10 mM). As seen from curve '*b*', upon the addition of hydrogen peroxide into PBS, an obvious catalytic characteristic appeared with an increase of the reduction current and a decrease of the oxidization current, which were ascribed to direct electron transfer between platinum nanozymes and the base electrode [2]. As control tests, we also investigated cyclic voltammograms of chitosan-modified GCE (*i.e.*, without PtNPs) in the presence and absence of hydrogen peroxide in PBS (pH 6.5, 10 mM). As indicated from the insets in Fig. S2-A, the peak currents were not almost changed before and after adding hydrogen peroxide to PBS (pH 6.5, 10 mM). These results in Fig. S2-A revealed that the reduction of hydrogen peroxide derived from platinum nanoparticles.


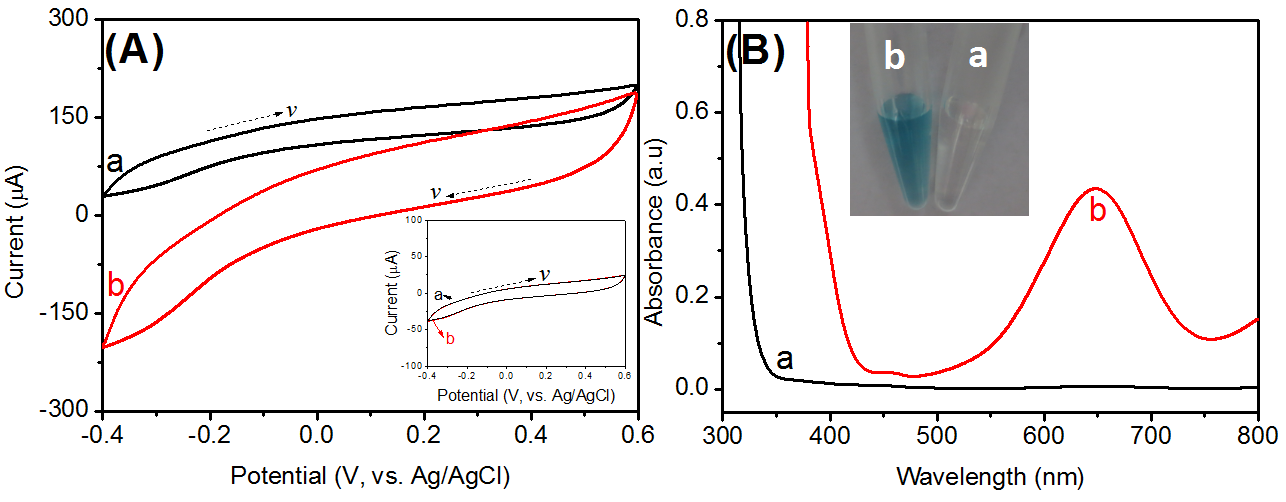


**Fig. S2** **A** Cyclic voltammograms of PtNP-chitosan-modified GCE in PBS (pH 6.5, 10 mM) without (a) and with (b) 0.5 μM hydrogen peroxide (H_2_O_2_) at 50 mV s^-1^ [insets: Cyclic voltammograms of chitosan-modified GCE in PBS (pH 6.5, 10 mM) without (a) and with (b) 0.5 μM H_2_O_2_ at 50 mV s^-1^]; **B** UV-vis absorption spectra of (a) TMB + H_2_O_2_ and (b) TMB + H_2_O_2_ + PtNP (insets: The corresponding photographs)

To further demonstrate that platinum nanoparticles had high peroxidase-like catalytic activity, we utilized UV-vis absorption spectroscopy to monitor PtNPs relative to 3,3′,5,5′-tetramethylbenzidine (TMB)-H_2_O_2_ system (Fig. S2-B). Typically, peroxidases, *e.g.*, horseradish peroxidase (HRP) and catalase (CAT), readily oxidize TMB into blue-colored molecules through H_2_O_2_ in aqueous solution. Curve '*a*' in Fig. S2-B gives UV-vis absorption spectroscopy of the mixture containing TMB and H_2_O_2_. Inspiringly, a new characteristic peak at 650 nm was achieved after mixing PtNPs, TMB and H_2_O_2_ (curve '*b*'), which originated from the oxidized product of TMB [3]. Moreover, a strong blue-colored product was appeared in the presence of PtNPs relative to TMB-H_2_O_2_ system (Fig. S2-B, insets, photograph '*b*' vs. photograph '*a*'), indicating the intrinsic peroxidase-like activity of platinum nanoparticles. Hence, PtNPs could be used as the peroxidase-like minics to catalyze the reduction of H_2_O_2_.

Logically, one question arises as to whether platinum nanoparticle (PtNP) could reduce hydrogen peroxide for the generation of the oxygen (O_2_). To realize our design, we prepared a homemade detection device for this purpose referring to the literature [4] (Fig. S3-A). Briefly, H_2_O_2_ aqueous solution (1.0 mL, 30 *wt* %) was first added into a glass container (capacity of 2 mL) that the top connected with a small hose. Another end of the hose was connected with a reservoir containing red indicator fluid. The reservoir inserted into a pipette through another small hose. All the connections were sealed with sealant. After PtNPs (500 μL, undiluted) was quickly injected into the container through the injection pipe, the pipe was clampped by a clip immediately. The volume change of liquid column in the pipette as a result of oxygen generation from the reduction of H_2_O_2_ was recorded. The whole process was operated at 25 ^o^C under ambient atmosphere. Photograph '*a*' in Fig. S3-B represents the initial signal of the liquid column in the pipette. When PtNP suspension was injected into the H_2_O_2_ aqueous solution, significantly, the red indicator liquid gradually moved ahead along the pipette (photograph '*c*'). Meanwhile, we also observed the bubble in the glass cup (photograph '*e*'). Obviously, the moving red indicator in the pipette was ascribed to the produced gas in the glass. By using an oxygen (O_2_) detector (http://www.dikaitech.com/product/16_183, GA50, DK Tech. Co., Ltd, Zhenzhou, China) (Fig. S3-C), we found that the as-produced gas was oxygen. For comparison, H_2_O_2_ aqueous solution was replaced by ultrapure water (*i.e.*, without H_2_O_2_), which was mixed with PtNPs. The red indicators did not move (Fig. S3-B, photograph '*b*'), and no bubble was produced in the glass cup (Fig. S3-B, photograph '*d*'). These results revealed that PtNPs could be used as the peroxidase-like mimics (*i.e.*, platinum nanozyme) to reduce H_2_O_2_ for generation of oxygen on the basis of the following equation ($H_{2}O_{2}\underset{\to}{Pt\mathrm{NP}}H_{2}O+O_{2}$).


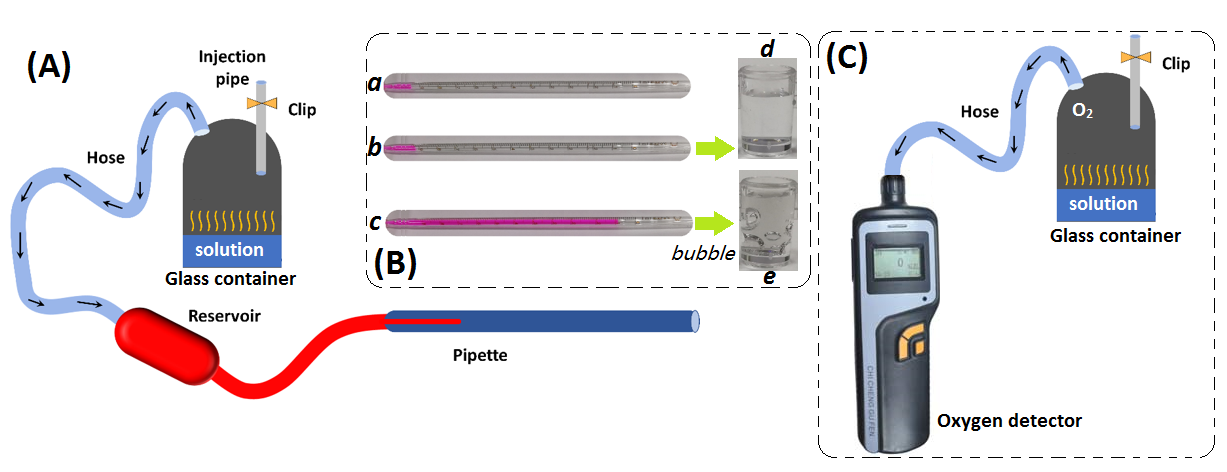


**Fig. S3** **A** Schematic illustration of homemade detection device; **B** photographs of the liquid column with the volume change in the pipette toward different components in the solution of glass container: (a) initial background signal, (b) ultrapure wate (1.0 mL) + PtNP, and (c) H_2_O_2_ aqueous solution (1.0 mL, 30 wt %) + PtNP (note: the corresponding photographs of glass containers); **C** schematic illustration of gas detection on a pump-suction oxygen (O_2_) detector by connecting the glass container

As is well-known, glucose oxidase (GOD) can oxidize glucose into gluconic acid and hydrogen peroxide in the presence of oxygen [5,6]. The as-produced hydrogen peroxide by GOD can be used as the substrate of platinum nanozyme, which can be reduced into H_2_O and O_2_. Further, the generated oxygen by platinum nanozyme can participate in the next cycle for glucose oxidase. The process can be simply summarized as the following reaction sequence:

At first GOD oxidized glucose molecules diffusing from the solution:

$glucose+ O_{2}\underset{\to}{\mathrm{GOD}} gluconic acid+ H_{2}O_{2}$ (1)

Then the produced H_2_O_2_ was reduced by platinum nanozyme into H_2_O and O_2_:

$H_{2}O_{2} \underset{\to}{\mathrm{PtNP}} H_{2}O+ O_{2}$ (2)

When glucose was present in the solution, the overall reactions for Eq(1) and Eq(2) could be fitted to the following reaction:

$\mathrm{glucose}\underset{\to}{GOD \& PtNP}$ gluconic acid + H_2_O (3)


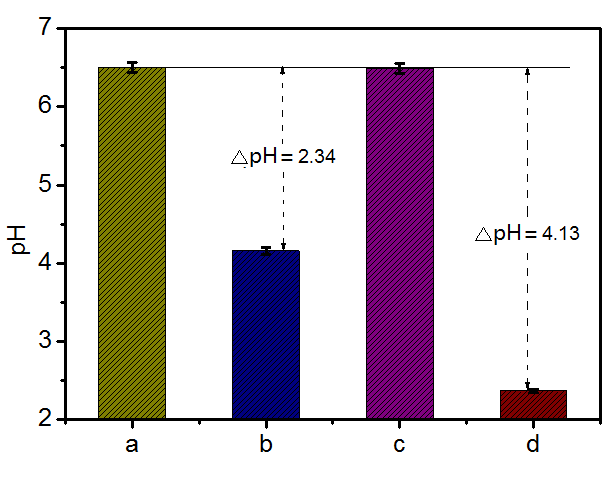


**Fig. S4** The pH values of **a** PBS (pH 6.5) containing 2.0 M glucose, **b** PBS (pH 6.5) containing 2.0 M glucose after reaction with GOD, **c** PBS (pH 6.5) containing 2.0 M glucose after reaction with PtNP, and **d** PBS (pH 6.5) containing 2.0 M glucose after reaction with GOD and PtNP (10 mM PBS used in this case). Each data point represents the average value obtained from three measurements, and the error bars stand for the 95% confidence interval of the mean for pH value

Typically, gluconic acid (2,3,4,5,6-pentahydroxy caproic acid, C_6_H_12_O_7_) is a non-corrosive, non-toxic, biodegradable, soluble, weak (p*K*_a_ = 3.86) organic acid. Generally speaking, gluconic acid solutions with different concentrations have various pH values. For example, 1.0 M of gluconic acid has a low pH value of ~1.93, whereas 0.1 M of gluconic acid exhibits a pH value of ~2.44. In this case, the free H^+^ ions by the produced gluconic acid molecules can change the pH of the detection solution (PBS, pH 6.5, 10 mM used in this work). If glucose (2.0 M used in this study) molecules were present in the detection solution, the produced gluconic acid by Eq(3) could offset the pH value of the buffer. To further elucidate this issue, pH values of PBS (100 μL, 10 mM, pH 6.5) containing 2.0 M glucose were monitored by a handheld pH meter in the presence of GOD or PtNPs (Fig. S4). As seen from column '*b*', the presence of GOD only cause a pH 2.34 variation of PBS (10 mM, pH 6.5) relative to background signal (column '*a*'), suggesting that GOD alone could result in a lower pH variation. In contrast, almost no pH change relative to background signal was observed in the presence of PtNPs (column '*c*' vs. column '*a*'), indicating that PtNPs could not catalytically oxidize glucose molecules. Significantly, a strong pH variation (~4.13, column '*d*' vs. column '*a*') was achieved when GOD and PtNPs were simultaneously present in PBS (10 mM, pH 6.5) containing 2.0 M glucose. The reason was attributed to the fact that the added glucose molecules in the detection solution were first oxidized to gluconic acid and hydrogen peroxide by the GOD in the presence of oxygen, and then the produced H_2_O_2_ was reduced to hydrogen oxide and oxygen *via* platinum nanozyme with peroxidase-like activity, thus pushing glucose oxidation forward to generate numerous gluconic acid molecules with the signal amplification.

**S2.3. Comparative Studies of Differently Labelling Strategies with mAb Antibody**

***Role of PtNP and GOD***: To elucidate the *in-situ* amplified capacity of using mAb-PtDEN-GOD for pH meter-based immunoassay, a comparative study was carried out for the detection of target CRP with a competitive-type immunoreaction on the CRP-coated microplates by using differently labelled probes, *e.g.*, mAb-DEN, mAb-PtDEN and mAb-DEN-GOD), respectively (Fig. S5). All immunoreactions were carried out on CRP-coated microplates by using 1.0 ng mL^-1^ target CRP as an example. The final pH measurements were implemented in 100 μL of PBS (10 mM, pH 6.5) containing 2.0 M glucose (10 mM). As shown from columns '*a*' and '*b*', the detectable pH values using mAb-DEN and mAb-PtDEN as the signal tags were almost the same as that of pH 6.5 PBS (10 mM), indicating that the labeled dendrimer and the encapsulated PtNPs in the dendrimer could not catalyze the oxidization of glucose to produce gluconic acid. When using mAb-DEN-GOD as the signal tag, however, a low pH variation (ΔpH ≈ 0.72) was achieved relative to pH 6.5 PBS (column '*c*'). The decreasing pH value was attributed to the conjugated GOD on the dendrimer towar the catalytic oxidization of glucose on the basis of the above-mentioned Eq(1). During this process, a few gluconic acid molecules were produced, thereby resulting in the decreasing pH of PBS. Significantly, the pH variation of using mAb-PtDEN-GOD (ΔpH ≈ 1.87 *vs*. pH 6.5, column '*d*') was more obvious than that of using mAb-DEN-GOD, indicating that the presence of PtNPs and GOD to the dendrimer could amplify the detectable signal of pH meter-based immunoassay. These results further revealed that platinum nanozymes could push the glucose oxidation forward to generate numerous gluconic acid molecules with the help of of GOD.


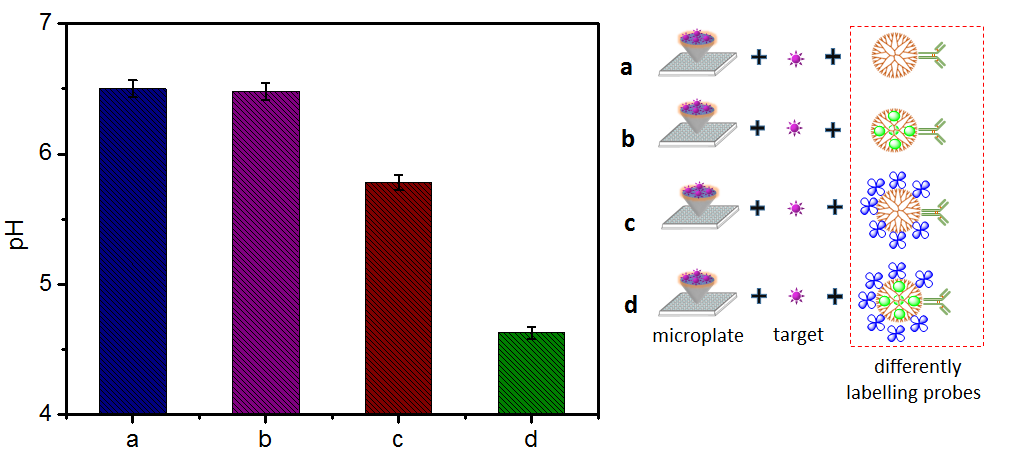


**Fig. S5** Comparative studies of pH meter-based immunoassay on the signal readout by using differently labelling probes: **a** mAb-DEN, **b** mAb-PtDEN, **c** mAb-DEN-GOD, and **d** mAb-PtDEN-GOD. All immunoreactions were carried out on CRP-coated microplates by using 1.0 ng mL^-1^ target CRP as an example. The final pH measurements were implemented in 100 μL of PBS (10 mM, pH 6.5) containing 2.0 M glucose. Each data point represents the average value obtained from three measurements, and the error bars stand for the 95% confidence interval of the mean for pH value

***Role of Dendrimer***: In the present work, bis-MPA-COOH dendrimer not only acted as a substrate for the encapsulation of PtNPs, but also enabled as the affinity support for the conjugation of GOD and mAb antibody. Trimethylol propane core-based bis-MPA-COOH dendrimer (generation 4) with a globular shape and branched structure was selected to have the merits of an organized monolayer and the ligand functionalization with high densitiy and accessibility. Moreover, bis-MPA-COOH dendrimer had 84 carboxyl surface groups, which was favorable for the functionalization with the biomolecules or proteins (www.sigmaaldrich.com/catalog/product/aldrich/806072?lang=zh&region=CN). Meanwhile, the dendrimer could provide a big room for the attachment of numerous PtNPs. To elucidate the role of dendrimers, a comparative study was executed by the same token for the detection of target CRP with a competitive immunoreaction on CRP-coated microplates by using differently signal tags including mAb-GOD, mAb-PtNP-GOD and mAb-DEN-GOD, respectively (Fig. S6). All immunoreactions were carried out on CRP-coated microplates by using 1.0 ng mL^-1^ target CRP as an example. The final pH measurements were implemented in 100 μL of PBS (10 mM, pH 6.5) containing 2.0 M glucose (10 mM). Thanks to the steric hindrance of mAb antibody, the labeled GOD molecules were limited. Usuaully, there is a ratio of 1 : 1 for enzyme and antibody. That is to say, one antibody can be labeled with one enzyme molecule. Relative to column '*a*', a very small pH variation (ΔpH = 0.27) was observed by using mAb-GOD as the signal tag (column '*b*'). As shown from columns '*c*' and '*d*', the pH variations could be improved by using mAb-PtNP and mAb-PtDEN-GOD, respectively. However, pH variation of using mAb-PtNP (column '*c*' vs. column '*a*') was less than that of using mAb-PtDEN-GOD (column '*d*' vs. column '*a*'). The reason might be most likely as a consequence of the fact that bis-MPA-COOH dendrimer could accommodate many PtNPs in the void, and provide more active sites for conjugation of biomolecules. From a statistical point of view, we might roughly estimate that one platinum nanoparticle with 20 nm in diameter could simultaneously conjugate 64 GOD or antibody biomolecules on the surface at most, while one bis-MPA-COOH dendrimer (generation 4) with a globular shape and branched structure could simultaneously conjugate 84 protein molecules [note: The calculation is based on the spherical surface area (*S*_NP_ = 4π*r_NP_*^2^) divided by the area of the GOD/mAb's radius-based circle (*S*_GOD/mAb_ = π*r_B_*^2^), where *r_NP_* stands for the radius of the nanoparticles and *r_B_* stands for the radius of the GOD or mAb (~5.0 nm in diameter)] [7]. Therefore, more GOD or mAb molecules could be simultaneously labeled onto the dendrimer than that of single platinum nanoparticle. Further, many platinum nanoparticles could be encapsulated into the dendrimer. When one antibody on the mAb-PtDEN-GOD reacted with the corresponding antigen, all PtNPs and GOD molecules attached to the dendrimer would be carried over and participate in the catalytic reaction. In this case, mAb-PtDEN-GOD would exhibit higher catalytic efficiency toward the substrate (glucose) than that of mAb-PtNP-GOD, thus resulting the signal amplification. Nevertheless, one disadvantage of using mAb-PtDEN-GOD is that the use of a macromolecule like the dendrimer added a strong value to the system relative to conventional GOD-labeled mAb antibody. In contrast, our system is simple, easy to operate without the requirements of expensive instrumentations and professional/technical personnel. Inspiringly, one-step immunoreaction with one-kind antibody reduces the assay time and decrease the assay cost on ~USD $1.93 for a single test with this system (~USD $7.81 per sample for cat# RAB0096 from Sigma-Aldrich; ~USD $10.454 for cat# ab260058 from Abcam).


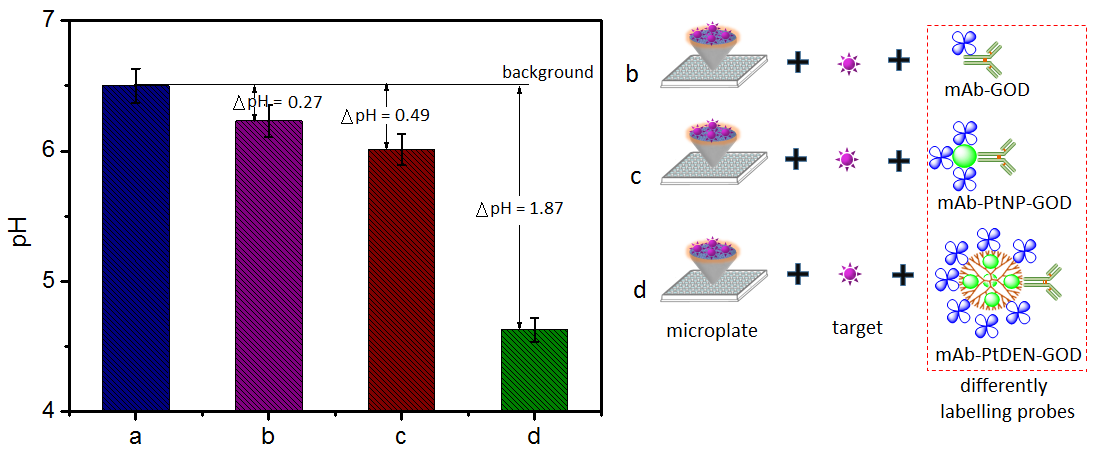


**Fig. S6** Comparative studies of pH meter-based immunoassay on the signal readout by using differently labelling probes: **b** mAb-GOD, **c** mAb-PtNP-GOD, and **d** mAb-PtDEN-GOD [note: **a** background signal of pH 6.5 PBS (10 mM)]. All immunoreactions were carried out on CRP-coated microplates by using 1.0 ng mL^-1^ target CRP as an example. The final pH measurements were implemented in 100 μL of PBS (10 mM, pH 6.5) containing 2.0 M glucose. Each data point represents the average value obtained from three measurements, and the error bars stand for the 95% confidence interval of the mean for pH value

**S2.4. Investigation of Substrate Effects**

As mentioned above, the pH variation of PBS (10 mM, pH 6.5) containing 2.0 M glucose derived from the conjugated GOD onto bis-MPA-COOH dendrimer toward catalytic oxidization of glucose, and the encapsulated PtNPs in the dendrimer toward the reduction of the produced H_2_O_2_ by GOD. To verify this point, the pH values of different substrates were measured on a handheld pH meter in the absence or presence of PtNPs (Fig. S7). Logically, one question arises as to whether the formed immunocomplexes between CRP and mAb antibody could casue the change of pH 6.5 PBS (10 mM). To verify this issue, a certain amount of CRP and mAb was initially added in pH 6.5 PBS (10 mM). Thereafter, pH values of the resultant solutions were determined. Column '*a*' gives pH value of in pH 6.5 PBS (10 mM). As shown from column '*b*', the formed immunocomplexes did not cause the pH change of pH 6.5 PBS (10 mM), indicating that the added proteins did not change pH of PBS. The secondary issue lies in the fact whether PtNPs could catalyze the gluconic acid (note: We purchased gluconic acid from Sigma, 49 – 53 wt % in H_2_O, cat# no.: G1951). We investigated pH values of gluconic acid before (column '*c*') and after (column '*d*') reaction with PtNPs. Obviously, the pH values of gluconic acid solution were almost the same in the absence and presence of PtNPs, suggesting that PtNPs could not react with gluconic acid. The third concern is whether H_2_O_2_ could be reduced by PtNP. As seen from column '*e*', 30 wt % H_2_O_2_ gave a weak acidic pH 5.9. After H_2_O_2_ mixed with PtNPs, however, pH changed to pH 6.8 (column '*f*'). More favorably, we could observe a large number of bubbles after addition of PtNPs in 30 wt % H_2_O_2_ (Fig. S7, outside, bottom photograph). Furthermore, we also utilized a pump-suction oxygen detector to determin the generated gas. The detectable signal on the oxygen (O_2_) detector heavily increased after incubation with the produced gas. These results revealed that PtNPs could reduce hydrogen peroxide (H_2_O_2_) for the production of oxygen gas, which was similar with previous reports [8-10]. As describe above, the generated oxygen gas could participate in the glucose oxidization by GOD. To further demonstrate the encapsulated PtNPs in the denderimer (PtDEN) with high peroxidase-like catalytic activity, bis-MPA-COOH dendrimers before and after encapsulation with PtNPs reacted with the mixture including TMB and H_2_O_2_, respectively. As seen from the insets in Fig. S7, a strong blue-colored product was appeared after the formation of PtDEN (photograph '*h*') relative to bis-MPA-COOH dendrimers alone (photograph '*g*'), suggesting the intrinsic peroxidase-like activity of PtDEN. Therefore, PtDEN could be used as the peroxidase-like minics to catalyze the reduction of H_2_O_2_. Based on the above-obtained results, we could make a conclusion that the added glucose molecules were initially oxidized to gluconic acid and hydrogen peroxide (H_2_O_2_) by the conjugated GOD, and then the produced H_2_O_2_ was reduced/consumed *via* platinum nanozyme with the peroxidase-like activity, thus pushing glucose oxidation forward to generate numerous gluconic acid molecules with the signal amplification.


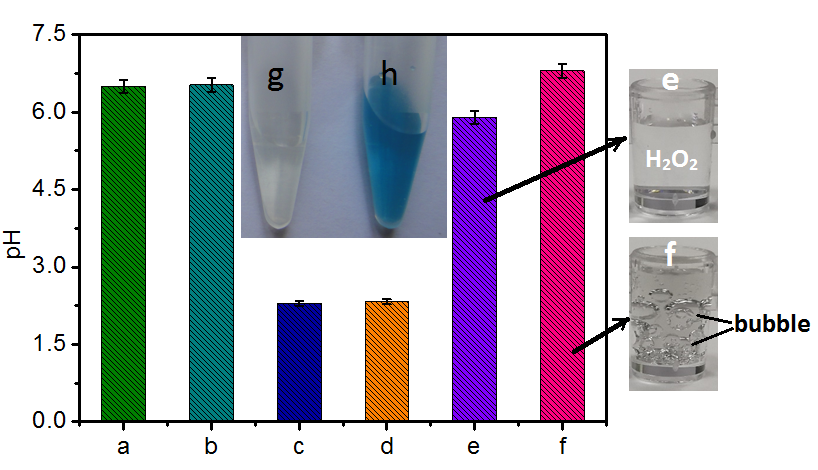


**Fig. S7** pH responses of different components: **a** pH 6.5 PBS (10 mM), **b** pH 6.5 PBS (10 mM) after incubation with 10 μg mL^-1^ CRP and 10 μg mL^-1^ mAb, **c** gluconic acid (2.0 mL, 2.0 M), **d** gluconic acid (2.0 mL, 2.0 M) after incubation with PtNPs, **e** 2.0 mL of 30 wt % H_2_O_2_ and **f** 2.0 mL of 30 wt % H_2_O_2_ after incubation with PtNPs (note: The photographs outside of Fig. were the corresponding real samples). Insets: Photographs of **g** bis-MPA-COOH dendrimers and **h** PtDEN after reaction with TMB and H_2_O_2_, respectively. Each data point represents the average value obtained from three measurements, and the error bars stand for the 95% confidence interval of the mean for pH value

**S2.5. Optimization of Experimental Conditions**

In this system, the detectable signal originated from the GOD and platinum nanozyme onto the dendrimer toward the cascade catalytic reaction of glucose molecules. Likewise the same as natural enzymes, the pH of PBS solution would directly affect the catalytic reaction of GOD and platinum nanozyme toward the substrates. Generally, the optimum pH of GOD is 5.0 ~ 6.0 for the oxidization of glucose. To simultaneously meet the catalytic efficiency of GOD and platinum nanozyme, we investigated the effect of pH of PBS on the signal of the immunoassay. As shown in Fig. S8-A, the pH variation initially increased with the increasing pH of PBS, and then decreased after pH 6.5. An optimum pH variation was achieved at pH 6.5. Higher or lower pH of PBS weakened the detectable signals of pH meter-based immunoassay. So, pH 6.5 of PBS was used for enzymatic reaction.

At this condition, the optimum molarity of pH 6.5 PBS buffer was investigated considering that this method was based on the measurement of H^+^ produced from GOD reaction. In this case, the buffering capability of PBS would hugely affect the pH variations. Therefore, we monitored the effects of different-molarity PBS (pH 6.5) on pH variation readout of pH meter-based immunoassay. As indicated from Fig. S8-B, an optimum pH variation was achieved at 10 mM with pH 6.5 PBS. A low molarity of PBS did not facilitate GOD enzymatic reaction. In contrast, protons generated from GOD enzymatic activity was favorable for production pH variation if the reaction was strongly buffered. Considering the concerns, we selected 10 mM PBS (pH 6.5) as the supporting electrolyte for development of pH meter-based immunoassay.

Also, we monitored the effect of molar ratio between mAb antibody and GOD on the signal of pH meter-based immunoassay since they were co-labeled on the dendrimer. Fig. S8-C gives the experimental results, and a maximum pH variation was observed at a molar ratio of 1 : 3 between mAb and GOD. The reason was attributed to the fact that the antigen-antibody conjugation probability depended on the labeled amount of mAb and GOD on the dendrimers, respectively. Although a high-concentration mAb on the dendrimer could decrease the immunoreaction time, it decreased the sensitivity of pH meter-based immunoassay. Considering this issue, a molar ratio of 1 : 3 for mAb : GOD was used to prepare the mAb-PtDEN-GOD.


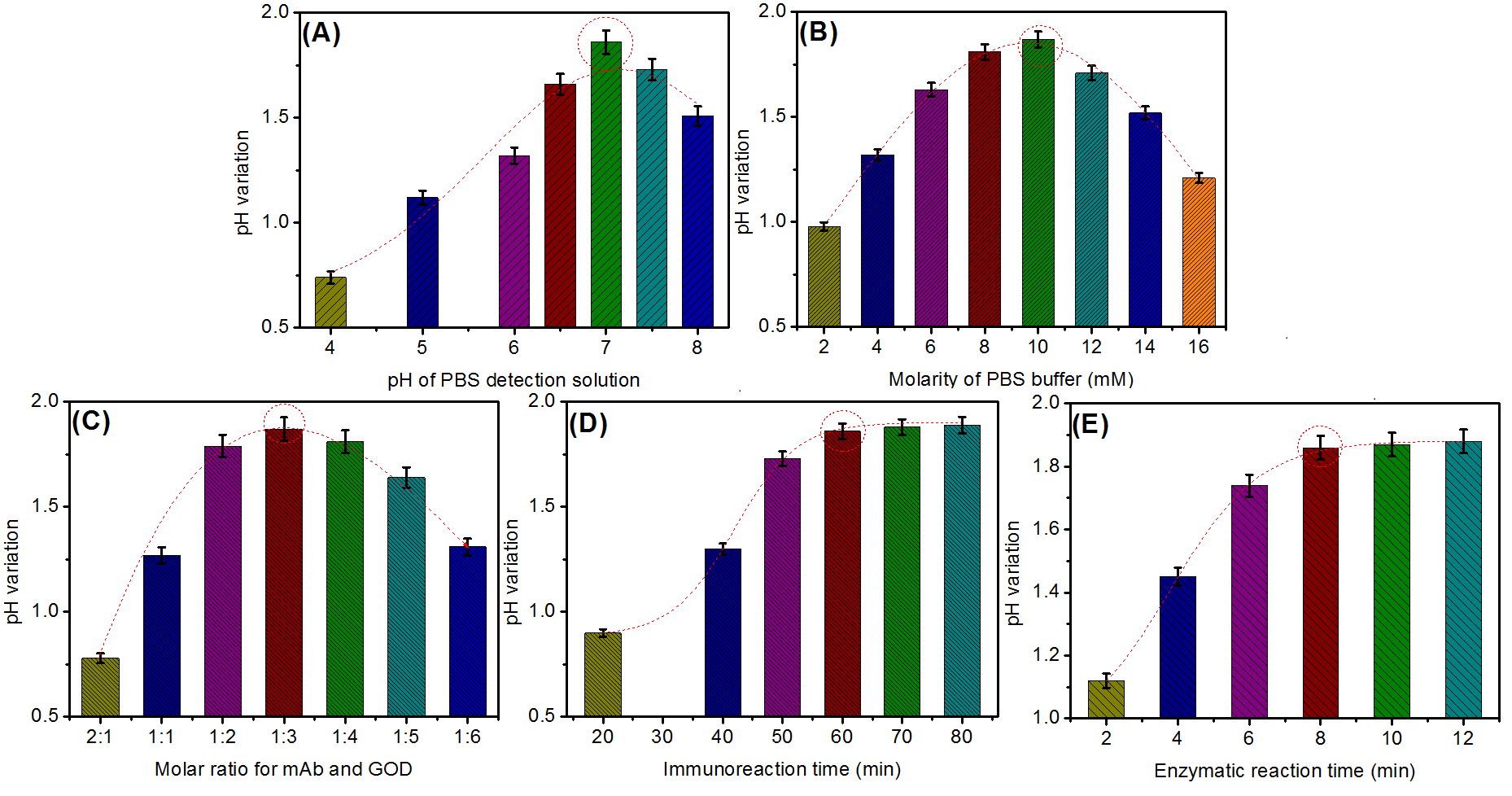


**Fig. S8** Effects of **A** pH of PBS buffer, **B** molarity of PBS buffer, **C** molar ratio (μM) between mAb antibody and GOD, **D** immunoreaction time between target CRP and the coated CRP on the microplate for mAb-PtDEN-GOD, and **E** enzymatic catalytic time of GOD/platinum nanozyme toward glucose molecules by using 1.0 ng mL^-1^ CRP as an example. Each data point represents the average value obtained from three measurements, and the error bars stand for the 95% confidence interval of the mean for pH variation

Generally, the antigen-antibody reaction and GOD catalytic reaction are perfectly implemented at close to normal temperature of the body (~37 ^0^C). To consider the detection convenience and future practical application, all immunoreactions and enzymatic reaction temperatures were carried out at room temperature (25 ± 1.0 ^0^C). In this regard, the effects of immunoreaction time and enzymatic reaction time toward glucose on the signal of the immunoassay was studied. Generally, it takes some time for the antigen-antibody reaction. In this case, we studied the effect of different immunoreaction times on pH variation of pH meter-based immunoassay between target CRP and the immobilized CRP protein on the microplate for mAb-PtDEN-GO. As shown in Fig. S8-D, pH variations relative to background increased with the increment of immunoreaction time, and reached a plateau after 60 min. To acquire an adequate pH signal, we selected 60 min as the immunoreaction time for target CRP. By the same token, the variation in the pH initially increased with the increasing catalytic time, and the tended to level off after 8.0 min (Fig. S8-E). To save the assay time, the steady-state pH variation was recorded at 8^th^ time for enzymatic reaction toward glucose.

**REFERENCES**

1. Li, J.; Yuan, R.; Chai, Y.; Che, X.; Li, W.; Zhong, X. Nonenzyme glucose sensor based on a glassy carbon electrode modified with chains of plaintum hollow nanoparticles and porous gold nanoparticles in a chitsan membrane. *Microchim. Acta* **2011**, *172*, 163-169.

(2) Chen, J.; Tang, J.; Yan, F.; Ju, H. A gold nanoparticles/sol-gel composite architecture for encapsulation of immunoconjugate for reagentless electrochemical immunoassay. *Biomaterials* **2006**, *27*, 2313-2321.

(3) Gao, Z.; Xu, M.; Hou, L.; Chen, G.; Tang, D. Irregular-shaped platinum nanoparticles as peroxidase mimics for highly efficient colorimetric immunoassay. *Anal. Chim. Acta* **2013**, *776*, 79-86.

(4) Wang, S.; Zhang, D.; Ma, Y.; Zhang, H.; Gao, J.; Nie, Y.; Sun, X. Aqueous solution synthesis of Pt-M (M = Fe, Co, Ni) bimetallic nanoparticles and their catalysis for the hydrolytic dehydrogenation of ammonia borane. *ACS Appl. Mater. Interfaces* **2014**, *6*, 12429-12435.

(5) Qiu, Z.; Shu, J.; Tang, D. Bioresponsive release system for visual fluorescence detection of carcino embryonic antigen from mesoporous silica nanocontainers mediated optical color on quantum dot-enzyme- impregnated paper. Anal. Chem. **2017**, 89, 5152-5160.

(6) Ren, R.; Cai, G.; Yu, Z.; Tang D. Glucose-loaded liposomes for amplified colorimetric immunoassay of streptomycin based on enzyme-induced iron(II) chelation reaction with phenanthroline. *Sens. Actuators B* **2018**, *265*, 174-181.

(7) Zhou, J.; Zhuang, J.; Miro, M.; Gao, Z.; Chen, G.; Tang, D. Carbon nanospheres-promoted electrochemical immunoassay coupled with hollow platinum nanolabels for sensitive enhancement. *Biosens. Bioelectron.* **2012**, *35*, 394-400.

(8) Gao, Z.; Ye, H.; Tang, D.; Tao, J.; Habibi, S.; Minerick, A.; Tang, D.; Xia, X. Platinum-decorated gold nanoparticles with dual functionalities for ultrasensitive colorimetric in vitro diagnostics. *Nano Lett*. **2017**, *17*, 5572-5579.

(9) Zeng, R.; Luo, Z.; Zhang, L.; Tang, D. Platinum nanozyme-catalyzed gas generation for pressure-based bioassay using polyaniline nanowires-functionalized graphene oxide framework. *Anal*. *Chem*. **2018**, *90*, 12299-12306.

(10) Pedone, D.; Moglianetti, M.; Lettieri, M.; Marrazza, G.; Pier, P. Platinum nanozyme-enabled colorimetric determination of total antioxidant level in saliva. *Anal. Chem.* **2020**, *92*, 8660-8664.
